# Enhanced Electroplasticity through Room-Temperature Dynamic Recrystallization in a Mg-3Al-1Sn-1Zn Alloy

**DOI:** 10.3390/ma14133739

**Published:** 2021-07-03

**Authors:** Hong Xu, Yu-Jie Zou, Yu Huang, Pin-Kui Ma, Zhi-Peng Guo, You Zhou, Yu-Peng Wang

**Affiliations:** 1 State Key Laboratory of Automotive Simulation and Control, Nanling Campus, Jilin University, No. 5988 Renmin Street, Changchun 130025, China; xh@jlu.edu.cn (H.X.); zouyj18@mails.jlu.edu.cn (Y.-J.Z.); huangyu17@mails.jlu.edu.cn (Y.H.); guozp18@mails.jlu.edu.cn (Z.-P.G.); zhouyou17@mails.jlu.edu.cn (Y.Z.); yupeng20@mails.jlu.edu.cn (Y.-P.W.); 2 Key Laboratory of Automobile Materials of Ministry of Education, School of Materials Science and Engineering, Nanling Campus, Jilin University, No. 5988 Renmin Street, Changchun 130025, China

**Keywords:** magnesium alloys, pulsed current, recrystallization, texture

## Abstract

It has been well known that electric pulse can be utilized to enhance the plasticity of metals, which is attributed to the change of dislocation dynamics, e.g., localized planar slip to homogeneous wavy slip. Here, we show another effect of pulse current, which facilitates texture weakening through room-temperature dynamic recrystallization and additionally improve the plasticity of a polycrystalline Mg-3Al-1Sn-1Zn alloy. By conducting a tensile test under electrical pulse, we found that the peak flow stress and fracture strain depend strongly on current density. As peak current densities increases, the flow stress drops and the fracture strain increases. Our Electron Backscatter Diffraction results suggest that dynamic recrystallization occurs at room temperature, which develops a weakened texture. Our work provides a new insight into electroplasticity mechanism in Mg alloys.

## 1. Introduction

Wrought magnesium alloys attract increasing attentions in lightweight structural applications because of their low density, high specific strength and stiffness, and good damping capacity [1,2,3]. However, the poor room-temperature ductility hinders the secondary processing, such as drawing and bending, thus, limiting their applications [4,5]. The electrically assisted manufacturing is a promising technology because the plasticity of metallic materials can be significantly enhanced by pulsed current during deformation. Meanwhile, the energy consumption due to pulsed current is usually lesser than that of heating material to a temperature that affords the same fracture strain, so that electrically assisted manufacturing is economically attractive [6]. As a consequence, this technology has been successfully applied to improve the formability of numerous metallic materials, i.e., aluminum alloys, magnesium alloys, titanium alloys, zirconium alloys, and steels [4,5,6,7,8,9].

However, the physical origin of electroplasticity is still not clear in the literature, due to the variety of different metallic materials. For example, Zhao et al. [10] recently reported the electroplasticity in a Ti-Al alloy originates from defect reconfiguration such as the transition of dislocation substructure from planner slip to wavy slip due to electropulsing. However, more significantly, the imposition of a pulsed current may lead to obvious microstructure modifications such as recrystallization, ageing, dissolution, and microstructure healing [10,11,12]. Jiang et al. [13] and Guan et al. [14] achieved enhanced high-temperature recrystallization in both cold-rolled AZ91 and AZ31 after subjected electropulsing treatment. Moreover, Conrad et al. [15] found that the pulsed current increased the recrystallization nucleation rate of the cold-worked copper. It has been proposed that the thermal effect caused by Joule heating of electropulsing treatment is mostly responsible for the enhanced recrystallization during deformation. In contrast, Park et al. [16] reported that recrystallization can occur at a lower temperature of 100 °C by electropulsing treatment, while it is difficult by traditional heat treatment in a furnace under the same temperature. Therefore, enhancing recrystallization by electropulsing treatment is not only a thermal response, but an athermal response is important. The above studies suggest strongly a correlation between recrystallization and electroplasticity. However, the effects of electric current on possible room-temperature recrystallization are still unclear in the literature. In particular, dynamical recrystallization mechanisms (DXR) during deformation assisted by pulse current has yet been well recognized.

In this work, electropulsing with different current densities was imposed to an Mg-3 Al-1 Sn-1 Zn (ATZ311) alloy during uniaxial tension at room temperature. The microstructure evolution and the recrystallization mechanism of ATZ311 alloy during electropulsing treatment were investigated in detail based on EBSD analysis. We show that dynamic recrystallization under pulsed current not only lowers the flow stress and accelerates recrystallization, but also refines grain and weakens texture. This work may provide a new insight to understand the dynamic recrystallization assisted by electropulsing in magnesium alloy.

## 2. Experimental Procedures

**Figure 1:**
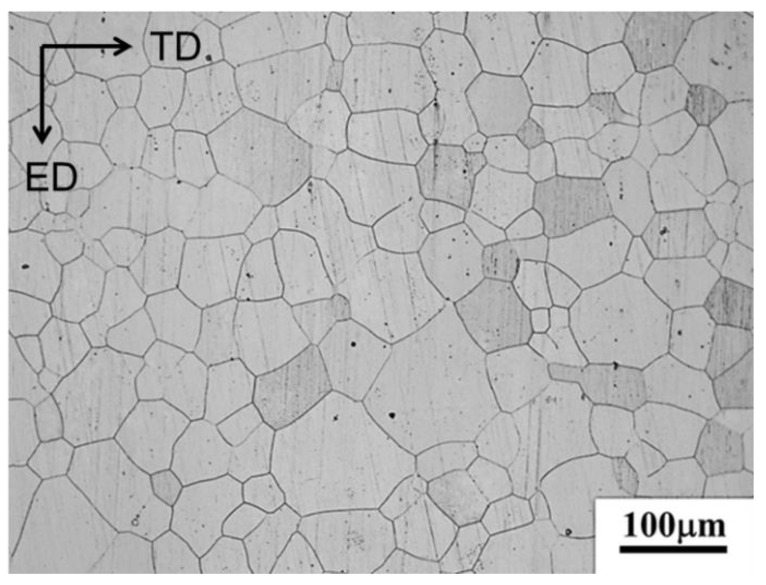
Optical microstructure of the initial alloy.

The extruded Mg-3 Al-1 Sn-1 Zn (wt. %), denoted as ATZ311 in this work, a sheet with a thickness of 1.2 mm was selected as raw material. Subsequently, the sheet was annealed at 200 °C for 1 h to relieve the residual stress, which acts as the initial alloy. The initial alloy has a near-equiaxed grain structure with an average grain size of ~43 μm (Figure 1).

**Figure 2:**
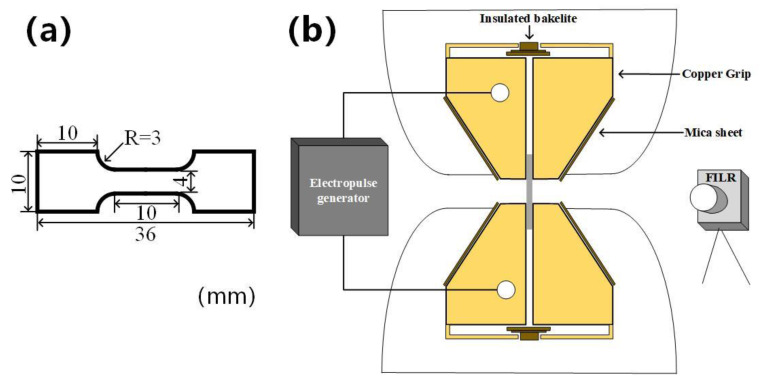
(**a**) Tensile specimen size; (**b**) schematic diagram of the electropulsed treatment assisted tension device.

The uniaxial tensile experiment under pulse current along extruded direction (ED) is performed on a WDW-200 tensile machine (CCKX, Changchun, China), with an initial strain rate of 10^−3^ s^−1^. Figure 2a shows the tensile specimen size, with a thickness of 1.2 mm. The pulsed current was generated by a 4000 FN DC power supply (CHANT, Zibo, China). The current parameters are shown in Table 1. Figure 2a shows the schematic diagram of electropulsing treatment assisted tension device where the pulse power is connected with the grips by wire. The grips and the tensile machine are separated by insulating wood and mica to ensure the insulation. Meanwhile, temperature variations of the sample during tension were recorded by FILR-A300 infrared thermal imager (FILR, Stockholm, Sweden) during the tensile test. Microstructure characterizations were carried out on the JEOL JSM-7001F field emission scanning electron microscope equipped (Jeol, Japan) with the electron backscattered diffraction (EBSD) analysis system. The EBSD microscope voltage and current are set as 20 V and 140–150 μA. We use CHANNEL5 software to process EBSD data. The samples for EBSD were prepared by electro polishing with an AC2 solution at 20 V for 60–120 s at −30 °C. The devices used for electropolishing are RXN-305D constant-voltage DC power supply (ZHAOXIN, Shenzhen, China) and HJ-3 temperature-controlled magnetic mixer (XUSHEN, Changzhou, China).

## 3. Results and Discussion

### 3.1. Mechanical Behavior during Tensile Test under Pulse Current

**Figure 3:**
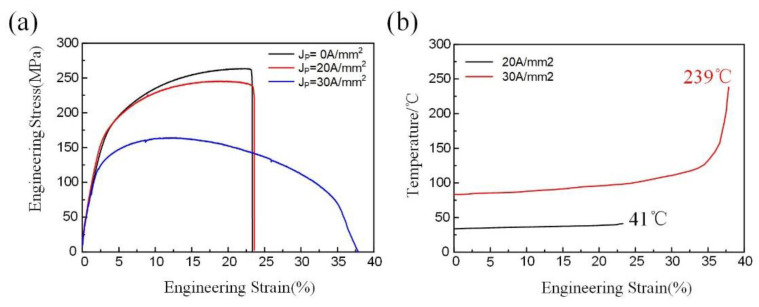
(**a**) Typical stress-strain curves; (**b**) temperature variations with strain curves under different peak current densities (J_P_).

Figure 3a shows the typical engineering stress-strain curves under different peak current densities (J_P_). It is clear that the flow stress drops but the fracture strain increases with an increase of J_P_, which is in complete agreement with the case of other metallic systems under pulse current [10,11,13]. The temperature change during tension was recorded by an infrared thermal imager, as shown in Figure 3b. The initial temperatures are ~30 °C and ~80 °C under 20 and 30 A/mm^2^, respectively, which indicates that increased peak current densities lead to upward initial temperatures. Specifically, under 20 A/mm^2^, the temperature change with increasing strain is very small, with a maximum temperature of only 41 °C, close to room temperature. Under 30 A/mm^2^, the temperature remains near constant before the strain of ~24%, but the temperature gradually increases until failure, with a maximum temperature of ~239 °C.

### 3.2. Microstructure Evolution and Dynamic Recrystallization Behavior during Pulse Current Tensile Test

At a strain of 8%, the material reaches ultimate tensile strength at 30 A/mm^2^. When the strain is 24%, the material has fractured at 20 A/mm^2^, while necking occurs at 30 A/mm^2^. So, the tensile tests are interrupted at a strain of 8% and 24% to investigate microstructure evolution in this work.

**Figure 4:**
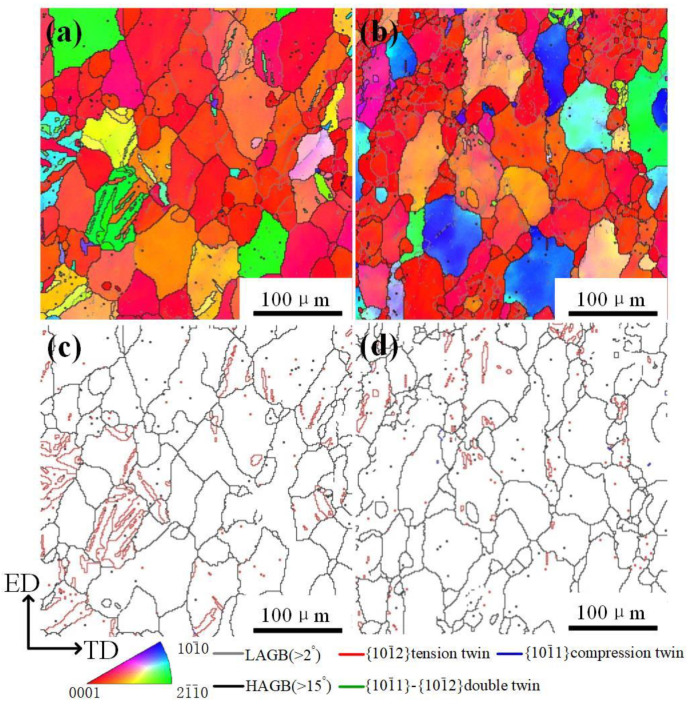
(**a**,**b**) IPF images and (**c**,**d**) twin distribution of (**a**,**c**) 8% strain and (**b**,**d**) 24% strain under 20 A/mm^2^.

Figure 4 shows EBSD analysis results of 8% and 24% and under 20 A/mm^2^. As noted earlier, this current density almost did not produce high temperature. Accordingly, this tensile deformation can be regarded as room temperature. As it is well known, both basal slip and {10–12} twinning is firstly activated during deformation. On the whole, from Figure 4, the deformed microstructure is analogous to the initial microstructure although some large parent grains are lightly elongated along the tensile direction. No obvious recrystallized structures are found. The significant deformation microstructure is characterized by several of {10–12} twins at 8%, but it just vanishes at 24%. This phenomenon is so-called to be detwinning [17].

At the initial stage, the formation of twins can hinder the dislocation slip because the twin boundary originating from the crystal reorientation between the twinned zones and surrounding zone within grain can effectively hinder dislocation slips, thereby resulting in a strong dislocation pile-up at the twin boundary [18,19]. As further straining, the slipped dislocation could across the twin boundary and penetrate into the entire twin. Meanwhile, here occur dislocation dissociation reactions from a perfect dislocation dissociating into a partial dislocation and a twinning dislocation.

Such a process offers a possibility for the nucleation of multiple twinning dislocations owing to the interaction of slipped dislocation with the twin boundary [20]. An avalanche-slip of multiple twinning dislocations will give rise to the reorientation of twins and the annihilation of twin boundaries. In reality, dislocation across the twin boundary needs to overcome a larger energy barrier. There is a hypothesis of electron wind force that the direct momentum transfers from the electron current to dislocations, which accelerates dislocation slip [10,11]. Consequently, the electron wind force effects might enhance the momentum of glide dislocations and promote dislocation across the twin boundary, possibly associating with the detwinning. The occurrence of such a detwinning would slow down strain hardening due to a decreasing overall resistance of twins to the dislocations slip after the vanishing of twins, as shown in Figure 3a.

**Figure 5:**
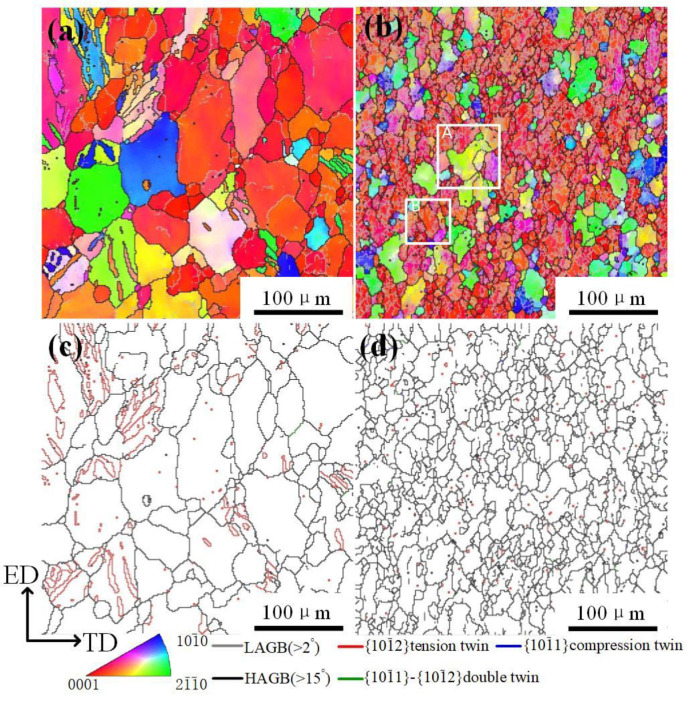
(**a**,**b**) IPF images and (**c**,**d**) twin distribution of (**a**,**c**) the initial sample and (**b**,**d**) the final sample under 30 A/mm^2^.

Figure 5 shows EBSD analysis results of 8% and 24% strain under 30 A/mm^2^. In the initial sample, there are a few {10–12} twins in the parent grains and still no recrystallized structure (Figure 5a,c), although the initial temperature over 80 °C, which seems to be similar to the case of 20 A/mm^2^. However, when the strain reached 24%, coarse parent grains are distinctly fined and amounts of low angle grain boundaries (LAGBs) existed within the parent grains. This indicates the occurrence of dynamic recrystallization (DRX) in this strain.

From Figure 3b, we can find that the temperature still decreases 100 °C at a strain of 24% under 30 A/mm^2^. At 100 °C, it is not enough to drive the occurrence of DRX, implying that electrical effect can significantly promote DRX, besides the thermal effects of the pulse current. Note that the DRX degree in different parent grains is different possibly due to orientation discrepancy [21].

According to an investigation on the influence of initial texture on the dynamic recrystallization in AZ31 alloy, it was shown that DRX takes place preferentially in those of grains favorable to the dislocation slips [22]. In addition, the straight twin boundaries in the initial stage have vanished, which suggests that twin boundaries can act as nucleation sites of DRX grains by constantly absorbing dislocations during the deformation process. The twin boundaries similar to high angle grain boundary can hinder the dislocation glide during the plastic deformation, and then dislocations pile-up at twin boundaries will rearrange themselves into dislocation wall or low angle grain boundaries to release energy, and this process leads to new DRXed grains formed in twin boundaries [23].

**Figure 6:**
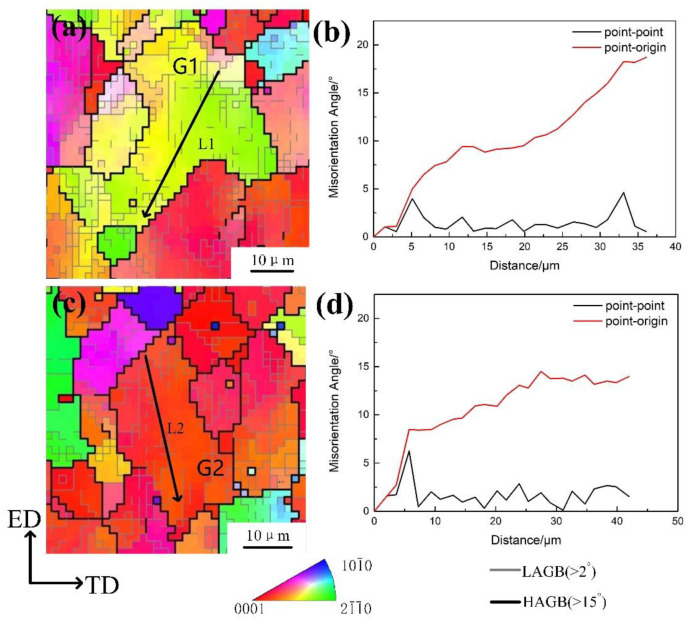
(**a**,**c**) the enlarged views of region A and region B in Figure 5b, (**b**,**d**) the misorientation distribution along L1 and L2, respectively.

We counted the misorientation distribution for more than 60 grains in Figure 5d. Representative area A and B are selected for illustration. Figure 6 shows the area A and B in Figure 5. The “point–point” is the misorientation of the data point with respect to the previous data point, and “point–origin” is the misorientation of the data point with respect to the starting point. It can be seen from Figure 6 that there is a large misorientation within the DRX grain, which can indicate that this LAGB must be caused through the lattice rotation due to basal slip within the DRX grains. Consequently, continuous DRX occurred within parent grains. As it is known, continuous DRX occurs by the cross-slip of <a> screw dislocations from basal plane to non-basal planes. According to the Friedel–Escaig mechanism [24], <a> screw can convert into an edge dislocation during cross-slip process [25]. Note that <a> screw dislocations can climb, thus abundant LAGB in parent grains could be as a result of dislocation rearrangements by cross-slip and climb. Zhao et al. [10] reported that pulse current can enhance cross-slip. Thus, more non-basal <a> dislocations could be activated and accumulated at LAGB. The LAGBs, which continuously absorb dislocations, can result in CDRX.

### 3.3. Texture Evolution during Pulse Current Tensile Test

**Figure 7:**
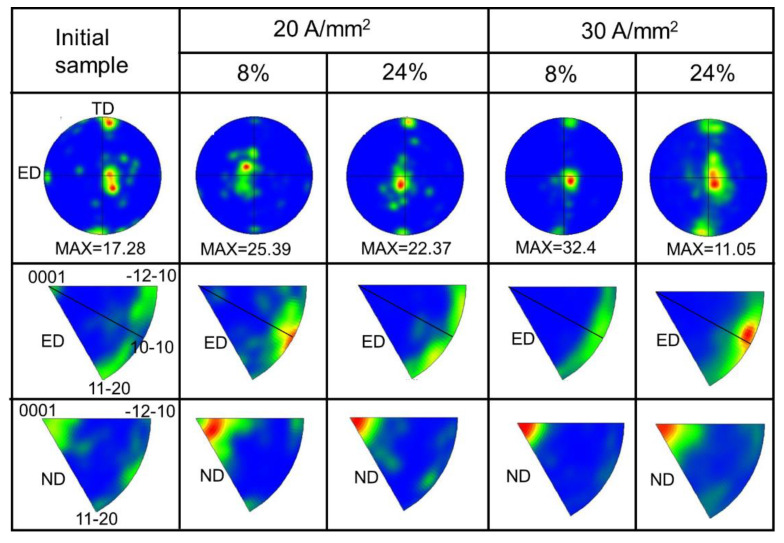
The (0001) pole figures and inverse pole figures of initial sample and deformed samples with different strains under different peak current densities showing texture evolution.

Figure 7 gives the (0001) pole figure and inverse pole figures of the initial sample and deformed samples with different strains under different peak current densities. Since the initial sample is from an extruded sheet, thus, it exhibits a triple basal texture: strong ND//(0001) called ND component, second TD//(0001) called TD component, and weak ED//(0001) called ED component via the careful analysis of (0001) pole figure and ND inverse pole figure. However, it should be noted that the intensity of ND component is higher than that of TD component. Additionally, {11–20} of majority grains is parallel to ED. With the strain increased to 8%, TD component weakens but ND component strengthens, and {11–20} transform into {10-10} being parallel to ED for both conditions, especially for case of 30 A/mm^2^.

During tension, those grains with ED component are in a favorable orientation for {10–12} twinning (see in Figure 4a and Figure 5a) due to basal planes perpendicular to the ED. {10–12} twinning will lead to the 86° lattice reorientation, namely *c*-axis rotates from being parallel to the ED to ND, which strengthens ND component. The start of tension deformation is accompanied by basal <a> slip, even for those grains with low Schmid factor of basal slips [26]. For the grains with ND and TD components, there is low activation energy of twinning and basal <a> slip, thus non-basal <a> slip will be activated after yielding [26]. Consequently, {11–20} transform into {10–10} being parallel to ED, which can be attributed to the continuous non-basal <a> slip [27,28].

With further strain to 24%, the texture intensity decreases in both conditions. Under 20 A/mm^2^, the reduction of the texture intensity can be attributed to detwinning because of no recrystallization in this case. Under, 30 A/mm^2^, apparent recrystallization occurred, which gives rise to an obvious decrease of the texture intensity. As mentioned earlier, twin boundaries provide nucleation sites, promoting DRX. It must be pointed out that further DRX at twin boundaries should weaken the twin texture caused by {10–12} twinning [29]. Meanwhile, the faster continued DRX process in the untwinned Mg matrix could result in the development of random texture, weakening the initial texture [30].

## 4. Conclusions

In this work, we systematically investigated the microstructure evolution of Mg-3Al-1Zn-1Sn alloy during uniaxial tension under pulsed current, during a room temperature dynamic recrystallization process. Our results show that the flow stress drops but the fracture strain increases with an increase of peak current densities. In the strain of ~8%, {10–12} twins are observed at both conditions, but no DRX. Due to {10–12} twins and non-basal slips, basal texture are strengthened and {11–20} is transformed into {10-10} being parallel to ED. Further strain to ~24%, detwinning occurred under 20 A/mm^2^, which could be attributed to gliding dislocations across twin boundaries under electron wind force, while significant DRX formed under 30 A/mm^2^. We proposed that electrical effect can significantly promote DRX except for the thermal effects of the pulse current because pulsed current only causes a low temperature of ~100 °C that is difficult to promote DRX. In this case, further DRX at twin boundaries and continue DRX should develop random texture, therefore, weakening initial texture. By investigating the room temperature dynamic recrystallization, our work may provide a new insight into electroplasticity mechanism in Mg alloys.

## Figures and Tables

**Table 1:** The current parameters.

	Voltage	Periodicity	Duration	J_P_
1	12 V	2000 μs	500 μs	20 A/mm^2^
2	12 V	2000 μs	500 μs	30 A/mm^2^

## Abbreviations

Abbreviation	Meaning
ATZ311	Mg-3Al-1Sn-1Zn
EDSD	electron backscattered diffraction
DRX	dynamical recrystallization mechanisms
CDRX	continue dynamical recrystallization mechanisms
ED	extruded direction
TD	transverse direction
ND	normal direction
J_P_	peak current densities
LAGB	low angle grain boundary
HAGB	high angle grain boundary
